# Cross-sectional study of gender differences in physical activity-related injuries amongst Chinese college students majoring in rehabilitation

**DOI:** 10.3389/fpubh.2022.912965

**Published:** 2022-09-08

**Authors:** Yanling Yu, Xian Li, Wangwang Yan, Beibei Feng, Jiadan Yu, Yuling Wang

**Affiliations:** ^1^Rehabilitation Medicine Center, The Sixth Affiliated Hospital, Sun Yat-sen University, Guangzhou, China; ^2^Department of Sport Rehabilitation, Shanghai University of Sport, Shanghai, China

**Keywords:** exercise, sports injury, risk-taking behaviours, injury incidence, young adults

## Abstract

The main objective of the paper was to explore the potential risk factors for physical activity-related injuries (PARI) amongst college students majoring in rehabilitation and to analyse gender differences. A random whole group sampling method was used to recruit freshmen to seniors aged 15–25 years from over 90 universities in China that offer rehabilitation. The total number of people included was 6,032, of which 1,989 were male and 4,043 were female. The underlying risk factors for PARI of different genders were assessed using a structured self-management questionnaire including sociodemographic characteristics, physical activity levels, risk-taking and protective behaviors, and PARI. Totally 6,032 questionnaires were obtained for final analysis, with 792 total number of injured persons (415 males, 377 females), the sum of the cumulative frequency of injuries to injured persons is 1,607 (881 males, 726 females) and a PARI risk of 0.27 (males: 0.44, females: 0.18; *p* < 0.001; sum of the cumulative frequency of injuries/total number of people surveyed/year). For male and female students, participation in sports teams, having a high level of PA as well as with antisocial behavior were risk factors for developing PARI. Regarding female students, regional differences was associated with elevated odds to suffer from PARI. The prevalence rates of PARI vary between male and female students. The research subjects were university students in rehabilitation. Compared to general college students, rehabilitation students have a certain knowledge base related to injuries, which defines the specificity and research value of this subjects. This study provides guidance for reducing PARI in students in rehabilitation and may provide a basis for developing future injury prevention mechanisms for university students in general.

## Introduction

Physical activity (PA) is any kind of physical movement that is performed through skeletal muscle contraction that requires energy expenditure. Active PA may decrease the risk of chronic non-communicable diseases (1, 2). Increased physical activity may lead to increased wellbeing in young people (3). Besides, active engagement in PA has been reported to improve physical fitness, such as VO_2_ max indicators (4).

The World Health Organization (WHO) global recommendation for healthy physical activity for adults is 150 min of moderate-intensity activity (or equivalent) per week. For adolescents, the recommendation is 60 min of moderate- to vigorous-intensity activity per day (5). According to the WHO, one in four adults and 81% of adolescents worldwide are physically inactive (not in line with the WHO global recommendations on physical activity for health) (6). Whereas in China, the problem of physical inactivity amongst adolescents is even more serious, with a physical inactivity rate of 84.3% (7). A global study shows that the inactivity rate among college students is about 41.4% (8). The level of physical activity among university students has declined in different countries (9). Physical inactivity is the fourth most important risk factor for the occurrence of chronic diseases worldwide and is associated with higher mortality rates for Chinese residents (10). Appropriate strategies are adopted to promote increased physical activity (11).

Currently, almost all countries and regions, including China, are involved in a global movement to promote physical activity (12). Prevention of physical activity related injuries (PARI) should also be on the agenda throughout the promotion of physical activity (13, 14).

Physical activity-related injuries generally refers to injury to the human body during PA. The PARI covered in this study is consistent with the concept cited by Mechelen in Sports Medicine, 1992, and adapted by Bloemers (14, 15).

In the short term, the fear of re-occurrence of previous physical activity-related injuries among college students ultimately leads to a decrease in physical activity participation (16, 17). PARI not only affect the academic performance of university students but are also detrimental to future social progress and development in the long run (18). In addition, PARI among university students have a more direct and indirect economic and social cost to families and society (19).

There are significant differences in PARI between male and female (20). Males report more PARI than females in all countries, but the extent of these gender differences varies considerably between countries (21).

Therefore, injury prevention should be targeted. According to the “prevention sequence” model, to develop appropriate prevention strategies, epidemiological surveys should first be carried out to determine the characteristics of the target population (15). Previous studies have disclosed some influential factors in PARI (22, 23).

This study focuses specifically on the group of university students majoring in rehabilitation, a group for which there is a great demand due to the limited number of rehabilitation students and the severely overrepresented population in need of rehabilitation. At the same time, given the specific curriculum structure, rehabilitation students have a certain knowledge base related to sports injuries compared to general college students, which makes this group uniquely valuable to study. The prevalence and characteristics of PARI in the group of university students majoring in rehabilitation are not well understood. Therefore, the aim of this study was to explore gender-related predictors of PARI among university students majoring in rehabilitation. This will also provide a basis for more in-depth development of injury prevention mechanisms for general university students in the future.

## Materials and methods

### Sampling

Random whole-group sampling was used to identify eligible schools by economic region (eastern, central, western, and north-eastern). College students majoring in rehabilitation from freshman to senior year were recruited between October 2020 and January 2021. Inclusion criteria were as follows: (a) college students majoring in rehabilitation, (b) those who signed the electronic informed consent form, and (c) those who completed ≥95% of the questionnaire.

### Data collection

This survey used a structured self-management questionnaire (Cronbach's coefficient alpha = 0.816). An electronic version of the questionnaire was administered to all students who signed the informed consent form by our trained staff, collecting relevant information on sociodemographic characteristics, PA levels, risk-taking behaviors and PARI and protective behaviors that occurred in the past year.

The demographic characteristics of the participants are summarized in Table 1. Demographic information includes age, grade, gender, family status, place of origin, weight and height, Near-sightedness, sports team membership, annual per capita household income and parental education level, etc.

**Table 1:** Sociodemographic contrast of PARI and non-PARI in different sexes of the population.

**Characteristics**	**Males (*****n*** = **1,989)**			**Females (*****n*** = **4,043)**		
	**PARI**	**Non-PARI**	**χ^2^/*t***	**PARI**	**Non-PARI**	**χ^2^/*t***
	**(*n* = 415)**	**(*n* = 1,574)**		**(*n* = 377)**	**(*n* = 3,666)**	
	***n* (%)**	***n* (%)**		***n* (%)**	***n* (%)**	
**Region**						
Eastern region	166 (20.6)	641 (79.4)	13.600**	191 (10.1)	1,708 (89.9)	10.716*
Central region	147 (25.3)	435 (74.7)		84 (11.1)	670 (88.9)	
Western region	92 (17.8)	426 (82.2)		87 (7.3)	1,113 (92.8)	
Northeast region	10 (12.2)	72 (87.8)		15 (7.9)	175 (92.1)	
**Grade**						
Freshman	142 (18.7)	619 (81.3)	5.319	149 (9.7)	1,384 (90.3)	0.852
Sophomore	119 (23.4)	390 (76.6)		98 (8.8)	1,010 (91.2)	
Junior	115 (22.4)	399 (77.6)		92 (9.0)	927 (91.0)	
Senior	39 (19.0)	166 (81.0)		38 (9.9)	345 (90.1)	
**Place of origin**						
Urban	170 (21.5)	619 (78.5)	0.368	167 (11.5)	1,280 (88.5)	13.092***
Rural	245 (20.4)	955 (79.6)		210 (8.1)	2,386 (91.9)	
**Body mass index (BMI) (kg/m** ^**2**^ **)**						
BMI <18.5 (underweight)	36 (14.5)	213 (85.5)	7.146	70 (7.1)	912 (92.9)	12.056**
18.5 ≤ BMI ≤ 23.9 (normal range)	268 (21.9)	953 (78.1)		252 (9.6)	2,380 (90.4)	
24.0 ≤ BMI ≤ 27.9 (overweight)	81 (21.4)	298 (78.6)		40 (12.6)	277 (87.4)	
BMI ≥ 28 (obese)	30 (21.4)	110 (78.6)		15 (13.4)	97 (86.6)	
**Near-sightedness**						
Yes	313 (21.4)	1,149 (78.6)	0.99	321 (9.6)	3,033 (90.4)	1.408
No	102 (19.4)	425 (80.6)		56 (8.1)	633 (91.9)	
**Only child**						
Yes	146 (20.6)	563 (79.4)	0.05	120 (11.7)	909 (88.3)	8.916**
No	269 (21.0)	1,011 (79.0)		257 (8.5)	2,757 (91.5)	
**Sports teams**						
Yes	128 (37.3)	37.30%	67.953***	86 (22.6)	295 (77.4)	87.307***
No	287 (17.4)	17.40%		291 (7.9)	3,371 (92.1)	
**Annual per capita household income**						
≤10,000	94 (18.1)	425 (81.9)	9.724*	96 (7.8)	1,127 (92.2)	7.132
10–50,000 (including 50,000)	166 (19.5)	684 (80.5)		160 (9.3)	1,563 (90.7)	
50–100,000 (including 100,000)	104 (24.9)	313 (75.1)		78 (10.7)	648 (89.3)	
>100,000	51 (25.1)	152 (74.9)		43 (11.6)	328 (88.4)	
**Mother's education level**						
Elementary school and below	155 (21.6)	564 (78.4)	2.478	119 (8.5)	1,277 (91.5)	7.847
Junior high school or vocational school	132 (20.4)	516 (79.6)		123 (8.6)	1,307 (91.4)	
High school or junior college	60 (18.5)	264 (81.5)		75 (10.8)	618 (89.2)	
Tertiary	36 (24.3)	112 (75.7)		35 (12.8)	238 (87.2)	
Bachelor's degree or above	32 (21.3)	118 (78.7)		25 (10.0)	226 (90.0)	
**Father's education level**						
Elementary school and below	96 (21.0)	362 (79.0)	2.591	76 (8.4)	827 (91.6)	4.795
Junior high school or vocational school	156 (20.3)	611 (79.7)		155 (9.4)	1,486 (90.6)	
High school or junior college	85 (20.8)	324 (79.2)		70 (8.5)	757 (91.5)	
Tertiary	45 (25.1)	134 (74.9)		39 (11.5)	300 (88.5)	
Bachelor's degree or above	33 (18.8)	143 (81.3)		37 (11.1)	296 (88.9)	
**PA level**						
Low	121 (16.0)	633 (84.0)	33.829***	94 (6.0)	1,474 (94.0)	40.869***
Moderate	97 (18.3)	432 (81.7)		144 (10.1)	1,284 (89.9)	
High	197 (27.9)	509 (72.1)		139 (13.3)	908 (86.7)	

* p < 0.05.

** p < 0.01.

*** p < 0.001.

The International Physical Activity Questionnaire (IPAQ) long form (24) assesses daily work, transportation, daily life, leisure exercise, and sedentary time, and calculates the level of PA that an individual engages in each week. The IPAQ has been validated to have a good validity and reliability in China (25, 26).

Participants were asked to complete 4 questions, which were questionnaires about their personal behaviors whilst engaging in PA. For example, (a) “Do you perform warm-up exercises before participating in physical activities?” (b) “Do you use protection when participating in physical activities?” (c) “Are you physically active in an appropriate environment?” (d) “Do you stretch or relax after participating in physical activities?” The 5-point Likert scale was provided for the responses, including “always,” “often,” “sometimes,” “hardly ever,” and “never”.

Risk-taking behavior refers to the choices individuals make in uncertain situations and with different tasks. It reflects the willingness of individuals to adopt behaviors that carries a significant degree of risk. That is, when individuals are faced with convergent conflict avoidance, they adopt risky behaviors in order to converge on a valuable or beneficial outcome that satisfies their needs. The Adolescent Risk-taking Questionnaire-Risk Behavior Scale (ARQ-RB) developed and revised by Zhang et al. (27), in China, with 17 revised items classified into four dimensions: stimulus-seeking behavior, reckless behavior, rebellious behavior and antisocial behavior (Cronbach's alpha = 0.734). Each item consists of five levels from one point to five points. The score of each risk-taking behavior factor is directly proportional to the willingness to participate in risk-taking behavior.

In addition, relevant information about PARI in the past 12 months was collected. PARI are generally injuries that occur during PA in humans. The PARI covered in this study are based on the concept mentioned by Mechelen in Sports Med in 1992 and adapted by Bloemers (14, 15) as any injury caused by physical education classes, sports, or recreational exercise with one or more of the following consequences: (1) the necessity to stop the current PA (sport), (2) the inability or inability to have a hand in the next planned PA (sport) overall, (3) the inability to attend class the next day and (4) the need to seek medical support. All participants were asked to report PARI based on the four criteria above, and a tally was finally performed. Those who had experienced PARI were asked to provide details of their most recent PARI.

### Processes and ethics

All institutions were required to ask student participants to accomplish the questionnaires within the same time. Before filling out the questionnaire, participants were asked to sign the informed consent form after reading the information sheet for the study and instructions for completing the survey. The return rate of the electronic questionnaire was 100%. A total of 6,710 questionnaires were collected, and 6,032 valid questionnaires were obtained after eliminating invalid questionnaires with regular or mixed answers, representing a valid response rate of 89.8%.

The study was approved by the Ethics Committee of the Sixth Hospital of Sun Yat-sen University (IEC Ref: E2020035).

### Statistical analysis

Categorical and continuous variables were presented with frequency (percentages) or means and standard deviations (SD). The discrepancies between the two groups, PARI and non-PARI, were tested by Pearson's chi-square test or independent samples *t*-test, respectively. A binary multivariate logistic regression analysis was conducted to screen the influences on males and females separately, using whether they were injured as the dependent variable and the statistically significant variables from the initial univariate analysis as independent variables. The data were analyzed using SPSS 26.0 software (IBM^®^ SPSS^®^).

## Results

### Sociodemographic characteristics

As shown in Table 1, in total, 6,032 participants were incorporated for analysis, including 1,989 males and 4,043 females, and their mean age was 19.82 years (SD = 1.43). In the entire sample, 792 students (13.1%) reported at least one PARI in the past year. There was a significant difference in the incidence of injury between males and females (χ^2^ = 155.652, *p* < 0.001), where the injury incidence was 20.9% (415/1,989) for males and 9.3% (377/4,043) for females. According to statistics, the sum of the cumulative frequency of injuries to the injured was 1,607 (males: 881, females: 726). The results showed that the overall risk of injury was 0.27 (sum of the cumulative frequency of injuries/total number of people surveyed/ year; males: 0.44, females: 0.18; *p* < 0.01).

The effect of demographic characteristics between males and females on PARI is shown in Table 1. For males, those with high levels of PA, in the eastern region, participation in sports teams and high annual per capita household income were associated with the occurrence of PARI. As for females, those with high levels of PA, in the eastern region, urban, obese, the only child, and those who participating in sports teams were more likely to report PARI (*p* < 0.05).

### Behaviors related to physical activity

Differences were observed between males and females in behavior related to PA. Males who almost never warmed up before PA were more vulnerable to develop PARI than those who sometimes warmed up (*p* < 0.05), as shown in Table 2.

**Table 2:** Contrast of PA related behaviors for PARI and non-PARI by gender.

**Physical activity (PA)-related behaviors**	**Males (*****n*** =**1,989)**			**Females (*****n*** = **4,043)**		
	**PARI**	**Non-PARI**	**χ^2^**	**PARI**	**Non-PARI**	**χ^2^**
	**(*n* = 415)**	**(*n* = 1,574)**		**(*n* = 377)**	**(*n* = 3,666)**	
	***n* (%)**	***n* (%)**		***n* (%)**	***n* (%)**	
**Doing warm-up**						
Always	112 (24.1)	353 (75.9)	12.211*	70 (10.6)	591 (89.4)	0.979
Often	116 (23.0)	388 (77.0)		100 (10.8)	825 (89.2)	
Sometimes	125 (18.0)	570 (82.0)		140 (8.5)	1,508 (91.5)	
Almost never	54 (21.4)	198 (78.6)		54 (7.9)	626 (92.1)	
Never	8 (11.0)	65 (89.0)		13 (10.1)	116 (89.9)	
**Use protective equipment**						
Always	53 (26.1)	150 (73.9)	6.629	15 (11.4)	117 (88.6)	6.635
Often	46 (24.6)	141 (75.4)		27 (17.0)	132 (83.0)	
Sometimes	92 (20.1)	365 (79.9)		63 (9.3)	612 (90.7)	
Almost never	102 (20.5)	395 (79.5)		113 (8.8)	1,166 (91.2)	
Never	122 (18.9)	523 (81.1)		159 (8.8)	1,639 (91.2)	
**Exercise in appropriate environment**						
Always	96 (24.1)	303 (75.9)	8.536	79 (10.5)	673 (89.5)	7.859
Often	156 (20.6)	602 (79.4)		134 (9.3)	1,308 (90.7)	
Sometimes	123 (18.1)	556 (81.9)		132 (8.6)	1,407 (91.4)	
Almost never	26 (27.7)	68 (72.3)		18 (8.0)	207 (92.0)	
Never	14 (23.7)	45 (76.3)		14 (16.5)	71 (83.5)	
**Stretch or relax**						
Always	91 (21.4)	334 (78.6)	6.03	80 (9.9)	728 (90.1)	6.166
Often	99 (22.2)	346 (77.8)		86 (8.2)	968 (91.8)	
Sometimes	123 (20.4)	481 (79.6)		119 (9.2)	1,174 (90.8)	
Almost never	73 (23.0)	245 (77.0)		77 (11.3)	605 (88.7)	
Never	29 (14.7)	168 (85.3)		15 (7.3)	191 (92.7)	

* p < 0.05.

### Risk-taking behaviors

The total score for risk-taking behavior was much higher in the PARI group compared with the non-PARI group. Higher scores were associated with the occurrence of PARI. By contrast, both in the PARI group scored significantly higher on antisocial behavior (*p* < 0.05), as shown in Table 3.

**Table 3:** Contrast of ARQ-RB scores of college students with PARI and non-PARI by gender.

**ARQ-RB factors**	**Males (*****n*** = **1,989)**			**Females (*****n*** = **4,043)**		
	**PARI**	**Non-PARI**	**t**	**PARI**	**Non-PARI**	**t**
	**(*n* = 415)**	**(*n* = 1,574)**		**(*n* = 377)**	**(*n* = 3,666)**	
	***n* (%)**	***n* (%)**		***n* (%)**	***n* (%)**	
Thrill-seeking behavior	3.29 ± 2.748	3.60 ± 3.632	1.918	3.64 ± 2.703	3.35 ± 2.697	−1.969
Rebellious behavior	2.30 ± 3.543	1.94 ± 3.645	−1.784	1.13 ± 2.268	0.79 ± 1.966	−2.764
Reckless behavior	0.22 ± 0.908	0.27 ± 1.064	0.814	0.07 ± 0.529	0.08 ± 0.548	0.337
Anti-social behavior	1.74 ± 2.307	1.44 ± 2.393	−2.337*	1.39 ± 1.824	1.02 ± 1.660	−3.789*
Total	7.55 ± 7.865	7.25 ± 8.96	−0.63	6.23 ± 5.508	5.25 ± 5.247	−3.449

* p < 0.05.

### Factors affecting males' PARI

The variables with significant differences in the above chi-square test or *t*-test were used to determine the odds ratios (ORs) and corresponding 95% CIs for PARI in logistic regression model. Non-athletic team members had a lower risk of PARI development (OR = 0.452, 95% CI: 0.346–0.591). High levels of PA were related to a greater risk of PARI compared with low PA levels (OR: 1.875, 95% CI: 1.443–2.436). In addition, high antisocial behavior scores were associated with elevated risk of PARI (OR = 1.069; Table 4).

**Table 4:** Risk factors for PARI amongst males.

**Variables**	**Partial regression coefficient (β)**	**Standard error (SE)**	**Odds ratios (ORs)**	**95% confidence interval (CI)**	***p*-Value**
**Sports teams**					
Yes			1		
No	−0.793	0.136	0.452	0.346–0.591	**<0.001**
**PA level**					
Low			1		
Moderate	0.265	0.154	1.303	0.964–1.762	0.085
High	0.628	0.134	1.875	1.443–2.436	**<0.001**
**Anti-social behavior**	0.067	0.023	1.069	1.023–1.118	0.003*
Constants	−0.723	0.163	0.485		**<0.001**

The bold values indicate a *p*-value of less than 0.01.

### Factors affecting females' PARI

As Table 5 shows, college students in the region of West China had a lower risk of developing PARI compared with the East (OR = 0.716, 95% CI: 0.546–1.587). Non-sports team members had a lower risk of developing PARI (OR = 0.353, 95% CI: 0.266–1.587). High levels of PA were related to a greater risk of PARI in a dose-dependent manner compared with low levels of PA (OR: 1.678–2.047). Individuals with high antisocial behavior scores had an increased risk of PARI (OR = 1.107, 95% CI: 1.048–1.587).

**Table 5:** Risk factors for PARI amongst females.

**Variables**	**Partial regression coefficient (β)**	**Standard error (SE)**	**Odds ratios (OR)**	**95% confidence interval (CI)**	***p*-Value**
**Region**					
Eastern region			1		
Central region	0.182	0.143	1.200	0.907–1.587	0.202
Western region	−0.334	0.139	0.716	0.546–1.587	0.008*
Northeast region	−0.159	0.284	0.853	0.489–1.587	0.576
**Sports teams**					
Yes			1		
No	−1.042	0.143	0.353	0.266–1.587	**<0.001**
**PA level**					
Low			1		
Moderate	0.517	0.140	1.678	1.274–1.587	**<0.001**
High	0.717	0.144	2.047	1.545–1.587	**<0.001**
**Anti-social behavior**	0.102	0.028	1.107	1.048–1.587	**<0.001**
Constants	−1.327	0.201	0.265		**<0.001**

The bold values indicate a *p*-value of less than 0.01.

## Discussion

This cross-sectional survey revealed that ~13% of college students majored in rehabilitation in China had suffered at least one PARI in the past 12 months, which was lower compared with the 22.7% prevalence rate in the previous study (28). In a survey on sports injuries among university students in Wuhan, the incidence of injuries among university students was 15.59% (29). In a survey of medical students, their lack of knowledge about physical health care led to a higher incidence of PARI (30). In contrast, the incidence of PARI was relatively low among university students in rehabilitation. The discrepancies in the rate of PARI found in this study could be related to the different study sample populations. Students in rehabilitation have background knowledge and skills in PARI concepts and related preventive measures (31).

In our study, males were at significantly higher risk of injury compared to females (0.44 vs. 0.18). Consistent with previous studies, different potential risk factors associated with PARI were observed between genders (14, 32). Males tend to be more actively involved in PA than females (6), which may increase their incidence of PARI. In addition, males are more likely to participate in competitive team sports such as basketball and football, which mostly involve high speed contact, jumping, sprinting, and spinning that are associated with common injury mechanisms (33). Given the gender-difference issue, the potential risk factors for the occurrence of PARI were experimentally explored separately by gender separately in our study.

Results of the study showed that the prevalence of PARI was higher in urban-dwelling females than those living in rural area (*X*^2^ = 13.092, *p* < 0.01). This may be explained by several factors between urban and rural residence, including economic level, awareness and habit of regular PA (34). The popularity of bike-sharing in cities has made urban students keen to get around by bicycle (35). Improvements in urban sports infrastructure, such as the creation of basketball courts and large squares, as well as public sports equipment, such as outdoor fitness equipment and sports facilities, have increased opportunities for students living in urban areas to participate in sports and leisure activities (36). To some extent, the above factors may be accounted for the different PARI rate between urban and rural dwelling students. Regarding the geographic and economic regional distribution, injury rates were much higher for both males and females in the eastern China, which may be due to differences in PA levels and awareness of injury prevention in each region (37).

Additionally, it is found that the rate of PARI was higher amongst members of sports teams than those non-sports team members, for both males and females (*p* < 0.05). This is partly because members of sports teams are usually required to attend regular training sessions and participate in different types of sports competitions, so the chances of PARI are higher (38).

For males, the higher the annual household income per capita, the greater the risk of PARI (Chi Square, same as above *x*^2^ = 9.724, *p* < 0.05). Those with higher household income are likely to be of middle-class or above social status and may place more emphasis on exercise and physical fitness. Students in a low-income family are less likely to be aware of the need for physical activity and afford popular sports, such as judo, gymnastics, tennis and so on (39).

In the current study, 29.1, 32.4, and 38.5% of the college students in rehabilitation responded engaged in high-, medium- and low-level PA, respectively. A significant correlation/association was observed between PA levels and the incidence of PARI in both males and females. Higher PA levels were associated with an increased risk of experiencing PARI, which echoed previous findings (40). The occurrence of PARI, in the short run, could place a negative impact on the motivation of college students to persevere in active PA due to fear of injury reoccurrence or aggravation (16, 17). In the long term, PARI may also increase the risk of other damage or disorders related to PA (41). Therefore, prophylactic measures to prevent PARI need to be advocated whilst promoting active PA engagement.

According to the findings, males who never warm up before exercise were less likely to be injured than those who always warm up. Several studies suggest that warming up is not associated with PARI (42), and more alternatives to warming up before exercise are more conducive to reducing PARI risk (43).

Risk-taking behaviors have been identified as a major contributor to PARI (44), and the results of this study showed that students with antisocial behaviors were at a higher risk for PARI (*p* < 0.05). Risk-taking behavior may be related to cognitive ability, which could explain why risk-taking behavior increases the risk of PARI (37). Poor awareness of risks also increases the odds of developing PARI (45).

In summary, this cross-sectional study had a relatively large sample size. It was possible to conduct a nationwide survey of rehabilitated college students in a low-cost electronic format. However, there are several limitations in the study. First, the cross-sectional design limited the findings of the study and no causal relationships could be drawn. In addition, the study was a self-administered questionnaire and did not include objective measures. Considering these limitations, longitudinal studies should be conducted in the future to expand the total sample size and sample specialty. Where necessary, measurement tools that objectively record physical activity could be used to avoid these limitations.

## Conclusions

Physical activity-related injuries is not an uncommon health issue amongst Chinese college students majored in rehabilitation. The prevalence rates of PARI vary between male and female students. Different risk factors were found associated with the occurrence of gender-specific PARI in the study. For male students, participation in sports teams, having a high level of PA as well as with antisocial behavior were risk factors for developing PARI. Regarding female students, sports team membership, a higher level of PA, with antisocial behavior, as well as regional differences, were associated with elevated odds to suffer from PARI. Our findings may play an important role in the development of physical activity-related prevention programmes for college students majoring in rehabilitation, with attention to differences between genders. In addition, they also play a more important role in promoting physical activity and physical activity-related injury prevention among college students in general majors. These results could increase young people's awareness of physical activity and physical activity-related injuries and better avoid sports injuries while promoting physical activity. Also, in the future prevention of physical activity-related injuries among general college students, different measures could be taken to address the gender differences in physical activity injuries.

## Data availability statement

The raw data supporting the conclusions of this article will be made available by the authors, without undue reservation.

## Ethics statement

The studies involving human participants were reviewed and approved by the study was approved by the Ethics Committee of the Sixth Hospital of Sun Yat-sen University (IEC Ref: E2020035). The patients/participants provided their written informed consent to participate in this study.

## Author contributions

YY and WY: conception of ideas and experimental design. YY and YW: data collection, analysis, and manuscript writing. LX, YW, FB, WY, and YJ: writing—review and editing. WY: editing and supervision. All authors contributed to the article and approved the submitted version.

## Funding

This study was supported by the grants from the Guangdong Hopson-Pearl River Education Development Foundation (Grant Number H20190116202012724).

## Conflict of interest

The authors declare that the research was conducted in the absence of any commercial or financial relationships that could be construed as a potential conflict of interest.

## Publisher's note

All claims expressed in this article are solely those of the authors and do not necessarily represent those of their affiliated organizations, or those of the publisher, the editors and the reviewers. Any product that may be evaluated in this article, or claim that may be made by its manufacturer, is not guaranteed or endorsed by the publisher.
